# Plyometric Training’s Effects on Young Male Karatekas’ Jump, Change of Direction, and Inter-Limb Asymmetry

**DOI:** 10.3390/sports12010001

**Published:** 2023-12-19

**Authors:** Alejandro Moreno-Azze, Estela Prad-Lucas, David Fandos Soñén, Francisco Pradas de la Fuente, David Falcón-Miguel

**Affiliations:** 1 ENFYRED Research Group, Faculty of Health and Sports Sciences, University of Zaragoza, 22001 Huesca, Spain; franprad@unizar.es (F.P.d.l.F.); dfalcon@unizar.es (D.F.-M.); 2 Faculty of Health and Sports Sciences, University of Zaragoza, 22001 Huesca, Spain; 717843@unizar.es (E.P.-L.); david.fandos.93@gmail.com (D.F.S.); 3 Faculty of Education, University of Zaragoza, 50009 Zaragoza, Spain

**Keywords:** between-limbs asymmetry, combat sports, explosive strength, kumite, stretch–shortening cycle, strength training

## Abstract

This study analysed the effects of performing a plyometric training programme on different types of jumping and specific changes of direction, and their respective asymmetries in karatekas. Twenty male karatekas (age 19 ± 4 years) were distributed in two groups, the control group (CG) and the experimental group (EG). The EG group (*n =* 10) performed a 6-week intervention of unilateral plyometric training, performing countermovement jumps (CMJ), drop jumps (DJ), and long jumps (SH). The tests performed at the beginning and at the end of the intervention were a unilateral and bilateral countermovement jump test (CMJ), single-leg hop test (SH), single-leg side-hop test (SSH), triple hop test (TH), and change of direction in a karate position test (MKUKS). The EG group obtained improvements in the CMJ with the stronger (*p* = 0.01; ES = 0.39) and weaker leg (*p* = 0.01; ES = 0.59), in the SH with the weaker leg (*p* = 0.01; ES = 0.45), in the SSH with the weaker leg (*p* = 0.03; ES = 0.33), in the MKUKS (*p* = 0.00; ES = 0.98), and improved the asymmetries obtained in the TH (*p* = 0.02; ES = −0.85). The GC group obtained significant differences in the CMJ with the stronger (*p* = 0.03; ES = 0.46) and weaker leg (*p* = 0.00; ES = 0.69), in the bilateral CMJ (*p* = 0.02; ES = 0.24), in the SH with the weaker leg (*p* = 0.00; ES = 0.34), in the TH with the stronger (*p* = 0.00; ES = −0.15) and weaker leg (*p* = 0.01; ES = 0.09), and in the MKUKS test (*p* = 0.04; ES = −0.94). A between-group analysis showed improvements of the EG over the GC in the TH with the stronger leg (*p* = 0.02; ES = 1.05). Performing plyometric training provides improvements in jumping, mainly in horizontal jumps, reducing inter-limb asymmetries in repetitive jumps.

## 1. Introduction

Sports such as boxing, judo, taekwondo, karate, and wrestling are considered combat sports [1]. Among combat sports, two groups of modalities stand out: those involving throwing, pinning, locking, blocking, and joint fighting, and others such as impact modalities involving punching, kicking, elbowing, and knee strikes [2]. In karate, there are different styles, known as ryu [3,4]. Within each of these styles, the modalities are divided into kata, which represents the traditional form of Karate, where pre-arranged movements are performed in the form of an exhibition, and kumite, which involves combat between two opponents, aiming to score points during the confrontation [5]. Karate is considered a high-intensity sport in which most offensive and defensive actions are performed with maximum speed and power effort [5], requiring high-intensity punching and kicking techniques before the opponent’s response. In kumite, unilateral dynamic defensive and offensive kinetic patterns are repeated, such as frontal, lateral, and circular kicks [6], and, additionally, karate athletes need to accelerate linearly and in different directions to attack and counterattack [7]. Due to this predominance of one limb over the other in different unilateral actions, among other possible factors, athletes may present asymmetries in their limbs [8,9,10]. The concept of asymmetries between limbs consists of comparing the performance or function of one limb with respect to the other [11]. Multiple factors are associated with greater asymmetries, such as gender, previous injury [12], and participation in a sport which emphasizes the use of a dominant limb [13]. Previous research suggests that an asymmetry greater than 10–15% may result in additional stress on one leg, ultimately leading to an increased risk of injury [8,13,14]. 

If we compare the injury risk of karate with other popular Olympic combat sports, karate has an injury risk similar to taekwondo but higher than that reported for judo and wrestling [15]. Increasing attention is being paid to the association between limb asymmetries in lower limb functional performance and sports injuries [14,16]. In relation to how injuries occur, injuries can be labelled as either contact or non-contact [17]. In the case of injuries which are caused by factors such as asymmetries, they are considered non-contact injuries, a category to which most injuries belong when studying the association between limb asymmetries in lower limb performance and sports injuries [17]. Asymmetries between the limbs may place the legs at greater risk of injury in sports, because the strong leg may endure excessive stress, due to over-reliance on that limb, and load, exerting a greater pressure, while the weaker side may not be able to effectively absorb the high forces associated with the sporting actions [16]. There are several articles demonstrating that asymmetries influence the likelihood of injury [14,16], mainly when it comes to hamstring injuries [18], anterior cruciate ligament injuries [19], and ankle sprains [20]. 

Injuries are a complex and multifactorial phenomenon, so it is advisable to have a thorough understanding of the risk factors discussed above in order to promote appropriate prevention strategies [21]. Due to all of the above, several studies have analysed this problem in recent years in combat sports such as karate, obtaining controversial results [22,23]. This is because it not only affects the probability of an increased risk of injury but also a decrease in performance in the sport practised [24]. An alteration in lower limb asymmetries such as through dynamic knee valgus is an important intrinsic factor in injuries [25]. However, some results are controversial, as some studies have examined the association between lower limb asymmetries and injuries and have shown that there is no association, as no significant relationship was observed between asymmetry and the incidence of future muscle injuries [26], fatigue, strength, and jumping tests [23]. On the other hand, an inverse relationship was demonstrated between the asymmetries that exist in the lower limbs, measured through the unilateral countermovement jump test (unilateral CMJ), and the specific performance of combat sports such as judo, measured through a specific test such as the Judo-Specific Fitness Test (SJFT), observing, as a conclusion, that possessing asymmetries between the lower limbs has a detrimental impact on the performance of specific tasks of each sport [27]. In karate, no significant differences (*p* > 0.05) between lower limbs tested through isokinetic dynamometry were observed [28]. 

Tests that analyse lower limb strength and power and observe asymmetries include unilateral jumps, including the one-legged CMJ, the triple jump, and the cross jump [29,30,31,32,33], and others that use the vertical jump with a bilateral drop [34]. In addition to jump tests, there are also studies that examine these asymmetries with isometric and isokinetic strength tests of knee extension [35], maximal strength in isometric hip adduction and abduction [36], maximum torque in isometric hip adduction [26], isokinetic knee extension and flexion, ankle plantar flexion and dorsal flexion, isokinetic dorsal flexion and plantar flexion [17,37], and isokinetic knee flexion and hip extension flexibility [14]. Other studies have used various strength tests and vertical, horizontal, and lateral jumps to assess asymmetries between limbs [38,39,40], due to the fact that asymmetries in power between limbs may be related to a reduction in jump height [41]. It had been shown that asymmetry of lean mass is a factor explaining asymmetry of strength and power during jumping, which implies a decrease in jump height, with results showing that an asymmetry of more than 10% in power results in a 3.5 inch performance deficit in jumping [41]. Vertical jumps involve closed kinetic chain movements with a stretch–shortening cycle, which have a greater similarity to movements performed in various sporting actions and are also more functional methods than some previous methods of assessing bilateral strength asymmetries such as isokinetic dynamometry, the isometric squat test, bilateral pedal strength, or the functional movement detection test [23]. Also, jumping tests are a viable method for quantifying limb asymmetries and are successfully used to prospectively identify limb imbalances [42], probably due to their similarity to the specific patterns performed in sport, their ease of implementation, and their time efficiency [40].

In relation to injury prevention, plyometric training is considered a suitable type of exercise to work on muscular strength and especially lower limb power [43], thereby reducing asymmetries and, thus, the risk of injury [44]. Plyometric training consists of fast and powerful actions involving muscle lengthening, followed immediately by rapid shortening of the same muscle [43]. It involves stretch–shortening cycle movements that involve a high-intensity eccentric contraction immediately following a fast and powerful concentric contraction [44]. These are explosive movements that generate a large amount of force quickly [44], and the type of actions performed during this type of work are explosive jumps [43]. Research indicates that plyometric training improves strength, power production, coordination, and athletic performance. Several studies on plyometric training have shown improvements in maximum strength whilst performing jumping exercises, which could be attributed to the improvement in coordination and the individual’s ability to rapidly increase muscle tension [44]. Another study [24] reported that in national and international level karatekas there were asymmetries measured through an horizontal jump test and a CMJ. And, they observed that bilateral training is a more effective method than unilateral training to improve CMJ asymmetry and that both unilateral and bipodal training are effective in reducing the percentage of asymmetries, being two forms of training suitable for preventing injuries and improving performance, as well as improving explosive strength. However, other studies have analysed the relationship between inter-limb asymmetries in jumping with the performance of karate-specific movements after plyometric training.

Therefore, the aim of the present study was to analyse the effects of a unilateral plyometric training programme on several types of jumping and specific changes of direction, as well as their respective inter-limb asymmetries, in young karatekas.

## 2. Materials and Methods

### 2.1. Experimental Approach to the Problem

This is a randomised controlled study design (A-B-B-A distribution), in which participants were allocated to one of the different groups according to their performance in pre-test assessments for a subsequent intervention. The participants were divided into two groups, the control group (CG) (*n =* 10) and the experimental group (EG) (*n =* 10). During the intervention period, the karatekas were encouraged to maintain their regular physical practice, while the EG added a unilateral plyometric training intervention. It was determined that the strong leg of each subject was the leg in which the highest number of tests obtained the best results. Data were taken before and after the intervention (pre-test and post-test, respectively). The data obtained from the pre-test in order to calculate its reliability were taken 2 weeks before the intervention, obtaining one set of data per test each week. Before starting the study, the subjects were familiarized with the technique of executing the exercises and the subsequent tests. Not exercising vigorously the day before and having their last meal at least 3 h before their scheduled test times were required.

### 2.2. Subjects

A total of 20 male karatekas (19.15 ± 4.91 years, 64.2 ± 13.23 kg and 170.0 ± 8.78 cm), practitioners of the karate kumite modality and belonging to a karate club from Zaragoza, voluntarily participated in the study. These participants had 10.95 ± 5.49 years of experience practising and competing in this sport in the Aragonese Karate Federation, and four of them, in turn, competed for the Spanish Karate Federation. These participants also had 1.95 ± 1.43 years of experience in strength training at the gym, which ranged from 5 years to 1 year. All the data were taken during the competitive period, between the sixth and eighth month. The subjects had a weekly training programme of four sessions per week (~2 h per day), combining strength/power and technical–tactical karate training. Before the investigation began, the participants and their legal representatives gave their written informed consent. The present study conformed to the recommendations of the Declaration of Helsinki and was approved by the institutional ethics committee.

### 2.3. Procedures

#### 2.3.1. Functional Performance Tests

##### Bilateral CMJ Test

The maximum bilateral vertical jump height in centimetres were calculated through the flight time, using the Optojump jump test instrument (Microgate, Bolzano, Italy) [5,45]. The test consisted of standing with both hands on the hips and one’s feet wide apart, followed by a downward movement, not standardized, and then a maximal-effort vertical jump [7,45]. All the participants were instructed to land upright and flex their knees after landing [7]. The subjects had three attempts, having 30 min of passive recovery between each attempt, and the best CMJ was selected for subsequent analysis.

##### Unilateral CMJ Test

The subjects were encouraged to jump as high as possible (Optojump, Microgate, Bolzano, Italy), standing on the test leg and swinging the free leg at the push-off if they needed to, provided it was flexed at a 90° angle from the hip and knee [5,45]. It was necessary to land steadily and carefully, holding the landing foot still for at least two to three seconds (any more hops or slips were disregarded). Between the different legs and jumps, there was a two-minute break for recovery. For additional analysis, the best three leaps from each leg were measured, yielding findings for the stronger (CMJ stronger) and weaker (CMJ weaker) legs.

##### Single-Leg Hop Test

One of the most popular methods for identifying differences in the capacity of the limbs to exert force in a horizontal direction was this test. The purpose was to jump as far as possible and measure that distance [39]. In its unilateral variant [39], it is regarded as a particularly accurate test for identifying asymmetries. From a standing position, on the leg with which the attempt was to be carried out, a jump was performed. With both hands behind the lumbar region, the participants had to jump as far as they could while maintaining balance and control, land on the same leg, and hold the landing foot still for at least two seconds [45]. The athletes were permitted to jump while swinging their unrestricted leg during push-off. The distance from the athlete’s most distal toe at the beginning of the test to their heel after landing was used to convert this measurement to centimetres. If a second jump or slide was performed after landing, the attempt was deemed invalid [45]. Each subject was given three tries with each leg, and the best result from each limb was selected. The recovery period lasted 30 s between jumps and 2 min between limbs. Results for the stronger (SH stronger) and weaker (SH weaker) legs were acquired in this manner, resulting in a more in-depth examination [45].

##### Single-Leg Side-Hop Test (SSH)

The jump consisted of standing sideways on the leg with which the attempt was to be carried out. Both hands were placed behind the lower back and the karatekas were required to jump as far as possible horizontally, sideways [45]. Landing was executed with the same leg with which the test had been performed, in the same starting position, and the free leg was allowed to support the other leg, as long as the test leg landed first. The free leg could be swung during the push-off phase of the jump [45]. The distance from the toe, in the starting position, to the nearest part of the foot at the end of the landing was measured in centimetres [45]. Recovery lasted 30 s between hops and 2 min between test legs. And, the best of the three attempts with each leg was chosen, obtaining the results of the stronger leg (SSH stronger) and the weaker leg (SSH weaker) [45] for further analysis.

##### Triple Hop Test (TH)

With their hands placed behind their lower back, standing on the test leg, the participants were instructed to hop as far as they could three times with the same leg [45]. The subjects were allowed to swing their free leg during the push-off phase and were instructed to land with control and balance, keeping the foot in position for two to three seconds. A further slide or jump was not permitted, otherwise the attempt would be deemed invalid [45]. The distance from the athlete’s most distal toe at the beginning of the test to their heel after landing was used [45]. The recovery time between jumps was 30 s and 2 min between legs. To obtain a more detailed analysis, the best of three attempts with each leg was measured, obtaining results for the stronger (strong TH) and weaker (weak TH) leg [45].

#### 2.3.2. MKUKS: Movement Change of Direction Test in a Karate Position

In a specific agility test, consisting of two parallel lines on the floor, 1.5 m long and 2 m apart, the participants were stood in a kumite fighting position in front of the first line, and, after the start signal, the karatekas moved as fast as possible in a karate-specific position (sori ashi), without crossing their legs, moving towards the second line [7]. When a subject stepped with their front foot on the second line, they stopped by turning 180 degrees and returned to the first line [7]. The movement was repeated three times. The results were taken using Witty photocells (Microgate, Bolzano, Italy), which recorded the execution time. Each participant tried the test three times, with a two-minutes recovery period between attempts, and the best performance was recorded for further analysis [7].

#### 2.3.3. Training Intervention

The plyometric training intervention lasted 6 weeks, with one session per week. This session was added either 48 h before or after a competition in which the subject in question had participated. A time range of 6 weeks of plyometric work was chosen because it was a duration that induces adaptations in the muscular power of the lower part of the body in karate competitors [3,5]. Before starting the intervention, the participants performed a standardized warm-up, which consisted of 5 min of continuous running, two sets of ten repetitions of bilateral squats, and two sets of five repetitions of unilateral squats, ending with dynamic stretching. The training programme consisted of three types of exercises performed unilaterally: a drop jump (DJ) from a height of 20 cm, CMJ, and SH. The intervention was programmed with a progressive increase in volume, with an increase in volume every one to two weeks, as shown in Table 1. In the training sessions, a rest break of 1 min was taken between exercises and of 3 min between sets.

The DJ exercise was performed from a raised surface, 20 cm above the ground, placing the hands on the hips or gripping the lower back. From the standing position, the subjects dropped the corresponding foot to the ground and, immediately upon touching the ground, jumped as far as possible, making sure that the reaction at the moment of touching the ground and jumping was as fast as possible. The CMJ consisted of jumping from a standing position, placing the hands on the hips or gripping the lower back. The participants were required to flex the corresponding leg by approximately 90 degrees (standardized depth) and then performed a vertical jump at maximum effort. The subjects were instructed to land upright and flex their knees after landing [7]. The SH exercise, starting from a unilateral position with the hands on the hips or clasped to the lumbar region, consisted of jumping horizontally from a static position, seeking to reach as far as possible. The participants were to be excluded if they failed to attend at least 80% of the intervention sessions.

### 2.4. Statistical Analyses

For the statistical analysis, the results were displayed with the mean ± standard deviation. The Shapiro–Wilk test was performed, with the aim of analysing the normality of the distribution of the data obtained. Thus, the related samples t-tests were used in the control and experimental groups to determine significant differences, establishing a value of *p* < 0.05 in the diverse variables. In order to eliminate potential pre-test differences, an ANCOVA was also performed for between-group comparisons using the pre-test as a covariable. In addition to the CV, a two-way random intraclass correlation coefficient (ICC) with absolute agreement and a 90% confidence interval was utilized to examine the reliability between sessions. To interpret the ICC values, Koo and Li’s (2016) earlier study was employed [46], where >0.9 = excellent, 0.75–0.9 = good, 0.5–0.75 = moderate, and <0.5 = poor, and the CV values were considered acceptable if <10% [47]. For the purpose of calculating the effect size (ES, 90% CI), the pooled pre-training SD in the chosen variables was used. The threshold values for Cohen’s d ES statistics were >0.2 (small), >0.6 (moderate), and >1.2 (large) [48]. For the within and between-group comparisons, the probabilities that the performance differences were better/greater, similar, or worse/smaller were determined. According to a qualitative assessment, the following quantitative probabilities of a positive or negative outcome were determined: 1%, most likely not; >1–5%, very unlikely; >5–25%, unlikely; >25–75%, possible; >75–95%, likely; >95–99%, very likely; and >99%, most likely [48]. The clinical effect was regarded as uncertain if the true value had a chance of being >25% beneficial and >0.5% harmful. If the benefit/harm odds ratio was 66, the situation remained ambiguous. The clinical inference was deemed to be positive when the odds ratio of benefit/harm was greater than 66. 

The statistical analysis tool used was IBM SPSS Statistics for Macintosh, Version 25.0 (IBM Corp., Armonk, NY, USA), as well as two specific Excel sheets, obtained from portsci.org (https://sportsci.org/index.html, accessed on 1 August, 2023). For the inter-limb asymmetries calculation, different equations were used [17]. In the present study, the following formula was used to calculate inter-limb asymmetries [49]:

100/maximum value (right and left) × minimum value (right and left) x − 1 + 100

## 3. Results

### 3.1. Subjects

No participants were excluded from further analysis after the intervention, according to the exclusion criteria. No athletes were injured during the unilateral plyometric training sessions. According to the results, eighteen out of twenty karatekas had the right leg as their dominant leg, eight of them belonging to the EG group and ten to the CG group, while two had the left leg as their dominant leg, both belonging to the EG group. However, sixteen participants had a dominant guard with their left leg, ten belonging to the EG group and six to the CG group, and four had a dominant guard with the right leg, all of them belonging to the CG group. Regarding which leg was considered the stronger leg, it can be observed that eight karatekas achieved better results with their right leg, considering it to be their stronger leg, three in the EG group and five in the CG group, while twelve had the left leg as their stronger leg, seven in the EG group and five in the CG group, due to the better results obtained with this leg.

### 3.2. Reliability Analysis

When taking the CV into account, all the tests displayed acceptable reliability, with all measurements displaying values less than 10%. All the tests displayed good reliability when the ICC data were taken into account (Table 2).

### 3.3. Within-Group Changes

The EG group obtained significant improvements in their CMJ stronger, CMJ weaker, SH weaker, and SSH weaker legs and in their Asy TH and MKUKS test results; they also obtained very likely improvements in their TH stronger leg and likely enhancements in their TH weaker leg, as showed in Table 3.

The CG group showed significant changes in their CMJ stronger, CMJ weaker, CMJ, SH weaker, TH stronger, and TH weaker legs and in their MKUKS test results, as well as likely improvements in their Asy SH test results, in their SSH weaker and TH weaker legs, and in their Asy TH test results (Table 3).

### 3.4. Between-Group Changes

**Figure 1:**
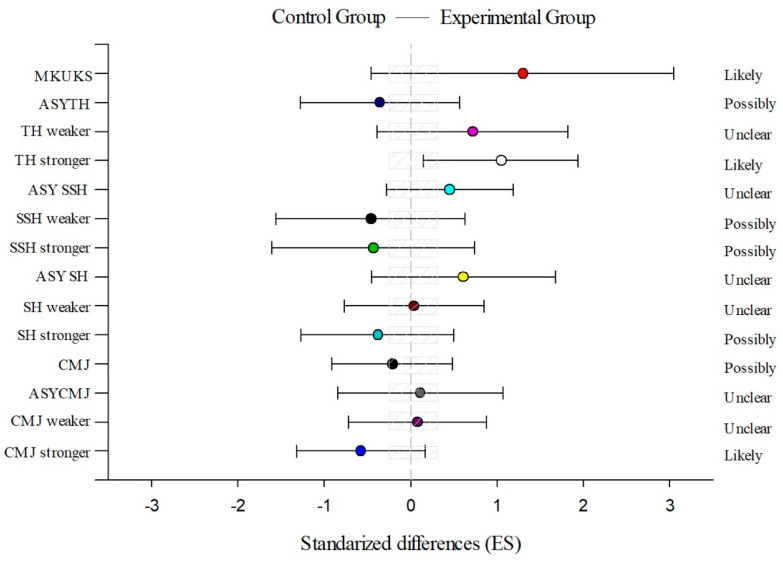
Experimental group’s plyometric training programme compared to the control group to improve a single-leg hop (SH) with the stronger and the weaker leg and the corresponding asymmetry (Asy SH), a single-leg side-hop (SSH) with the stronger and the weaker leg and the corresponding asymmetry (Asy SSH), a triple hop (TH) with the stronger and the weaker leg and the corresponding asymmetry (Asy TH), a bilateral countermovement jump (CMJ), a single-leg countermovement jump (CMJ) with the stronger and the weaker leg and the corresponding asymmetry (Asy CMJ), and a change of direction in a karate position (MKUKS). The bars indicate uncertainty in the true mean changes with 90% confidence limits. Trivial areas were the smallest worthwhile change (see the “Methods” section).

Figure 1 shows likely improvements in the MKUKS test (ES = 1.30) and significant enhancements in the TH stronger test (*p* = 0.02; ES = 1.05), while no statistically significant differences are found in the rest of the variables (Table 4).

## 4. Discussion

The main purpose of this study was to analyse the effects of a plyometric training intervention on jumping and a specific change of direction and their respective asymmetries in young karatekas. The main findings obtained were as follows: (1) significant improvements were found in the CMJ stronger, CMJ weaker, and SH weaker legs and in the SSH weaker, TH stronger, and TH weaker legs after the plyometric training programme; (2) the EG group showed very likely and significant enhancements in their MKUKS test results. Likely and very likely improvements were obtained in the TH stronger leg and MKUKS test results in the EG group compared to the CG group.

In the CMJ, the results were slightly higher (ES: 0.24 to 0.69) than those obtained in another study where they carried out a plyometric training intervention lasting 6 weeks (ES: 0.04 to 0.35) [24]. However, it should be noted that the results obtained in the present study showed better effects (ES: 0.24 to 0.69) compared to both unilateral (ES: 0.04 to 0.20) and bilateral (ES: 0.09 to 0.35) training [24]. They also reflect results in line with those obtained in a study where bilateral and unilateral CMJs executed with the weaker and stronger legs were improved (ES: 0.50 to 0.85) in the group that performed a unilateral plyometric training programme [50]. All these results suggested that performing a unilateral training programme may improve vertical jump, whether in a unilateral or bilateral direction. Also, performing combined vertical and horizontal plyometric exercises might obtain better effects in vertical jumps than performing only one exercise in the training programme. On the other hand, the above-mentioned research analysed vertical jumping performance in team sports players [50]. Therefore, the analysis of vertical jumping after plyometric training in combat sports is warranted in future research. 

Similar results were obtained in the SH test (ES: 0.24 to 0.45), in a comparison with a study on karatekas (ES: 0.29 to 0.47) [24], highlighting better results in unilateral plyometric training compared to bilateral plyometric training. Both studies agree with recent research noting that plyometric training can significantly increase the performance of horizontal jumps, which may be due to the fact that it has a significant effect on hip and thigh power [51]. With respect to the between-group differences, it should be noted that the unilateral plyometric training group showed more improvements in the SH (ES: 0.25 stronger leg and 0.45 weaker leg) than group CG (0.24 and 0.34, stronger and weaker leg, respectively).

As mentioned above, previous studies have shown that training protocols vary in the number of weekly sessions (2–3 sessions/week) and in the number of weeks of intervention (between 4 and 16 weeks), sets (between two and six), and repetitions (between two and twelve). Thus, comparing differences between studies is complicated, given the difficulty involved in finding relationships between them. Therefore, it seems likely that training volume may be a variable to be taken into account. 

Despite the fact that research suggested significant changes and specific adaptions after plyometric training in which issues such as jumping direction and force application [52] were considered, there is scarce information on the performance of various horizontal hops and single-sided hop. All of this is in accordance with the characteristics of combat sports, in which there is a predominance of horizontal movements in multiple directions coupled with different unilateral technical actions [7,8,9,10]. Considering this, it might be interesting to analyse the effects of unilateral plyometric training by looking at cyclic or acyclic jumps. A study with young elite fencers analysed the explosive and reactive strength by means of repeated vertical hopping contact time and height after a plyometric training programme, without any significant enhancement [53]. In this study, likely and very likely improvements (ES: 0.38 to 0.40) were found in the TH, both with the stronger and weaker leg, while likely enhancements (ES: 0.33) were achieved in the SSH’s weaker leg in group EG. Likewise, likely changes (ES: 1.05) were achieved when the EG group and the CG were compared. 

It had been noted that performing a training programme on one side is an acceptable strategy to reduce asymmetries between the legs [52,54]. Recent studies have shown changes in asymmetries between strength and jump tests, suggesting that the asymmetry is both test- and metric-specific [55]. Regarding the finding of significant differences in the level of asymmetry, it was observed that they had been reduced after the intervention, obtaining significant improvements in the TH (ES: −0.85, *p* = 0.02). This could be very positive given that karate actions can be predominantly horizontal. With respect to the between-group comparison, statistically significant improvements were found in the TH (ES: −0.36) in the CG group, while no significant differences were found in the rest of the variables. However, it seems likely that these improvements may be due to an improvement in the subjects’ coordination or an improvement in neuromuscular activation; so, future research is warranted.

Recent research with young male basketball players highlighted improvements in actions where COD was involved [52]. Along the same lines, statistically significant improvements were found in the COD test in the karate position (ES: 0.98, *p* = 0.00), a similar result to those shown in other studies [56,57] in which tests which influence agility and changes of direction are used in sports other than karate. The intervention of the unilateral plyometric training program was predominantly on the frontal plane and combined horizontal and vertical force vectors. However, the displacement in the karate-specific COD presented on a lateral plane and a horizontal force vector. This is why studies of force vectors similar to the COD-specific tests are warranted. 

This study had some restrictions that require addressing. Firstly, it would be interesting to analyse the effects of unilateral plyometric training in terms of force vectors and applied force. Secondly, as it is a unilateral type of training, analysing the differences between the effects of the different variables depending on the leg with which the training is started, for example, whether it is a strong leg or a weak leg, could be warranted in future research. Furthermore, further research could be carried out to compare the effects of various unilateral plyometric interventions on inter-limb asymmetries in additional training subjects with diverse ages, genders, and combat sports, as well as their related impacts on jumping and direction change.

## 5. Conclusions

The implementation of the unilateral plyometric exercise programme contributed significant improvements in the CMJ stronger, CMJ weaker, and SH weaker legs and in the SSH weaker, TH stronger, TH weaker legs. Moreover, very likely and significant enhancements in the MKUKS results after the plyometric training programme were observed. Likely and very likely improvements were obtained in the TH stronger leg and in the MKUKS test in the EG group compared to the CG group.

## Figures and Tables

**Table 1:** Unilateral plyometric training programme.

	DJ		CMJ		SH	
	Sets	Repetitions/Leg	Sets	Repetitions/Leg	Sets	Repetitions/Leg
Session 1	2	4	2	4	2	4
Session 2	3	4	3	4	3	4
Session 3	4	4	4	4	4	4
Session 4	4	6	4	6	4	6
Session 5	4	8	4	8	4	8
Session 6	4	8	4	8	4	8

**Table 2:** Measures of pre-intervention reliability in the MKUKS and functional performance tests (n = 20).

TEST	Difference (90% CL)	TEM (90% CL)	CV (90% CL)	ICC (90% CL)
CMJ	−0.44 (−0.76; −0.13)	0.64 (0.52; 0.85)	2.43 (1.96; 3.23)	0.99 (0.99; 1)
CMJ stronger	−0.05 (−0.07; −0.03)	0.04 (0.03; 0.05)	0.28 (0.22; 0.37)	1 (1; 1)
CMJ weaker	−0.1 (−0.25; 0.05)	0.31 (0.25; 0.4)	1.64 (1.32; 2.18)	1 (1; 1)
SH stronger	1.08 (−0.66; 2.83)	3.53 (2.86; 4.68)	1.78 (1.44; 2.37)	0.99 (0.98; 1)
SH weaker	−0.71 (−2.52; 1.1)	3.66 (2.96; 4.85)	2.16 (1.74; 2.87)	0.99 (0.98; 0.99)
SSH stronger	0.04 (−1.88; 1.96)	3.88 (3.13; 5.14)	3.29 (2.65; 4.38)	0.97 (0.94; 0.98)
SSH weaker	−1.38 (−3.23; 0.48)	3.74 (3.02; 4.96)	2.96 (2.39; 3.94)	0.97 (0.94; 0.98)
TH stronger	−1.88 (−5.25; 1.5)	6.82 (5.52; 9.05)	1.41 (1.14; 1.87)	1 (0.99; 1)
TH weaker	−3.29 (−7.17; 0.59)	7.84 (6.34; 10.4)	1.6 (1.29; 2.12)	1 (0.99; 1)
MKUKS	0.37 (0.18; 0.57)	0.4 (0.32; 0.52)	6.27 (5.04; 8.39)	0.91 (0.82; 0.95)

Note. SH = single-leg hop with the stronger and the weaker leg; SSH = single-leg side-hop with the stronger and the weaker leg; TH = triple hop with the stronger and the weaker leg; CMJ = countermovement jump bilaterally and with the stronger and weaker legs; MKUKS = movement change of direction test in a karate position; CL = confidence limit; TEM = typical error of measurement; CV = coefficient of variation expressed as a percentage of TEM; ICC = intraclass correlation coefficient; difference = difference in the mean between the two trials.

**Table 3:** Performance changes and asymmetries after unilateral plyometric training.

	Experimental Group (*n* = 10)					Control Group (*n* = 10)				
	Pre-Test	Post-Test	ES	Outcome	*p*	Pre-Test	Post-Test	ES	Outcome	*p*
CMJ stronger (cm)	13.24 ± 6.41	15.34 ± 4.35	0.39 (0.12; 0.67)	likely	0.01 *	16.71 ± 3.3	18.42 ± 2.94	0.46 (0.18; 0.74)	likely	0.03 *
CMJ weaker (cm)	11.81 ± 5.21	15.39 ± 4.83	0.59 (0.32; 0.85)	very likely	0.01 **	16.07 ± 2.74	18.47 ± 3.55	0.69 (0.29; 1.09)	very likely	0.00 **
Asy CMJ %	9.11 ± 8.99	0.12 ± 8.56	0.69 (−2.79; 4.17)	unclear	0.45	3.01 ± 9.58	−1.06 ± 17.35	−2.77 (−9.42; 3.89)	likely #	0.06
CMJ (cm)	25.95 ± 9.02	28.35 ± 9.18	0.26 (0.1; 0.42)	possibly	0.19	33.11 ± 6.27	34.79 ± 6.31	0.24 (−0.04; 0.52)	possibly	0.02*
SH stronger (cm)	165.44 ± 43.69	175.56 ± 37.62	0.25 (0; 0.51)	possibly	0.36	188.45 ± 21.07	193.91 ± 17.64	0.24 (−0.14; 0.62)	unclear	0.11
SH weaker (cm)	156 ± 37.48	173.89 ± 33.43	0.45 (0.25; 0.64)	very likely	0.01 **	184.73 ± 21.82	193.18 ± 18.07	0.34 (0.02; 0.66)	likely	0.00 **
Asy SH %	5.06 ± 9.51	0.47 ± 6.85	0.15 (−0.5; 0.8)	unclear	0.27	9.96 ± 27.22	0.32 ± 3.82	0 (0; 0)	likely	0.19
SSH stronger (cm)	131.11 ± 19.97	131.33 ± 24.84	−0.02 (−0.38; 0.33)	possibly	0.48	153.09 ± 12.55	155.45 ± 13.87	0.16 (−0.25; 0.57)	unclear	0.96
SSH weaker (cm)	122.89 ± 22.45	132 ± 26.02	0.33 (0.02; 0.65)	likely	0.03 *	147.45 ± 12.35	157.36 ± 14.59	0.69 (0.2; 1.17)	likely	0.09
Asy SSH %	6.61 ± 4.91	−0.61 ± 6.15	−1.10 (−1.72; 19.51)	unclear	0.08	3.43 ± 7.3	−1.53 ± 8.68	−0.08 (−0.85; 0.69)	possibly #	0.06
TH stronger (cm)	496.67 ± 114.1	545 ± 113.61	0.38 (0.2; 0.56)	very likely	0.28	607.73 ± 79.89	593.64 ± 74.47	−0.15 (−0.41; 0.12)	possibly #	0.00 **
TH weaker (cm)	489.89 ± 116.88	540.56 ± 102.33	0.40 (0.18; 0.62)	likely	0.46	589.55 ± 77.98	597.27 ± 75.91	0.09 (−0.13; 0.31)	likely	0.01 *
Asy TH %	1.49 ± 4.02	0.46 ± 4.33	−0.85 (−4.12; 2.41)	unclear	0.02 *	2.98 ± 2.5	−0.65 ± 3.74	0.56 (−0.21; 1.32)	likely	0.65
MKUKS (s)	5.91 ± 1.47	4.84 ± 0.89	0.98 (0.35; 1.6)	very likely	0.00 **	4.92 ± 0.86	4.12 ± 0.38	−0.94 (−1.27; −0.61)	most likely #	0.04 *

Note. SH = single-leg hop with the stronger and the weaker leg; Asy SH = asymmetry in the single-leg hop; SSH = single-leg side-hop with the stronger and the weaker leg; Asy SSH = asymmetry in the single-leg side-hop; TH = triple hop with the stronger and the weaker leg; Asy TH = asymmetry in the triple hop; CMJ = bilateral countermovement jump and with the stronger and the weaker leg; Asy CMJ = asymmetry in the unilateral countermovement jump; ES = effect size; CL = confidence limit, * *p* < 0.05, ** *p* < 0.01. # denotes a harmful effect. All the results are presented in the same direction; that is, a positive change is considered an improvement, while a negative change is considered an impairment.

**Table 4:** Efficiency of the unilateral plyometric training compared to the CG.

CG vs. EG			
	ES	Outcome	*p*
CMJ stronger (cm)	−0.58 (−1.32; 0.17)	likely	0.32
CMJ weaker (cm)	0.08 (−0.72; 0.88)	unclear	0.67
Asy CMJ %	0.11 (−0.84; 1.07)	unclear	0.91
CMJ (cm)	−0.21 (−0.91; 0.49)	possibly	0.95
SH stronger (cm)	−0.38 (−1.27; 0.5)	possibly	0.27
SH weaker (cm)	0.04 (−0.77; 0.85)	unclear	0.57
Asy SH %	0.61 (−0.45; 1.68)	unclear	0.91
SSH stronger (cm)	−0.43 (−1.61; 0.74)	possibly	0.69
SSH weaker (cm)	−0.46 (−1.56; 0.63)	possibly	0.66
Asy SSH %	0.45 (−0.28; 1.19)	unclear	0.93
TH stronger (cm)	1.05 (0.15; 1.94)	likely	0.02 *
TH weaker (cm)	0.72 (−0.39; 1.82)	unclear	0.17
Asy TH %	−0.36 (−1.28; 0.57)	possibly	0.65
MKUKS (s)	1.3 (−0.46; 3.05)	likely	0.16

Note. SH = single-leg hop with the stronger and the weaker leg; Asy SH = asymmetry in the single-leg hop; SSH = single-leg side-hop with the stronger and the weaker leg; Asy SSH = asymmetry in the single-leg side-hop; TH = triple hop with the stronger and the weaker leg; Asy TH = asymmetry in the triple hop; CMJ = bilateral countermovement jump and with the stronger and the weaker leg; Asy CMJ = asymmetry in the unilateral countermovement jump; ES = effect size; CL = confidence limit, * *p* < 0.05. All the results are presented in the same direction; that is, a negative change is considered an improvement by the CG group.

## Data Availability

The data presented in this study are available on request from the corresponding author. The data are not publicly available due to patient privacy concerns.
